# Ascites Secondary to Spontaneous Bladder Rupture: A Case Report

**DOI:** 10.7759/cureus.79369

**Published:** 2025-02-20

**Authors:** Isabel Bessa, Ana Costa, Elisabete Ribeiro, Letícia Marques Leite

**Affiliations:** 1 Internal Medicine, Hospital da Senhora da Oliveira, Guimarães, PRT; 2 Internal Medicine, Hospital de Braga, Braga, PRT

**Keywords:** acute kidney injury, ascites, bladder rupture, emergency department, urology

## Abstract

Ascites is a common finding in clinical practice, typically originating from hepatic, malignant, cardiac, renal, or infectious diseases. However, other less frequent etiologies must be considered, making the medical history a critical component in establishing the diagnosis. Acute kidney injury is also frequently seen in the emergency department, arising from diverse causes, some of which may overlap with those of ascites. Late-onset bladder rupture is a rare but serious complication following pelvic radiation therapy. Delayed presentation can lead to diagnostic challenges and life-threatening consequences if unrecognized. We present the case of a 51-year-old female, with a history of cervical cancer treated with chemoradiotherapy 15 years earlier, who presented with progressive abdominal pain, distension, dysuria, and oliguria. Examination revealed a distended abdomen with a palpable fluid thrill. Laboratory findings showed acute kidney injury, leukocytosis, and metabolic acidosis. Initial imaging identified ascites and an empty bladder. After urinary catheterization drained over three liters in one hour, suspicion of bladder rupture arose. A contrast-enhanced CT cystogram confirmed the diagnosis. The patient underwent laparotomy for bladder repair. This case highlights the diagnostic challenge of spontaneous bladder rupture and the importance of considering this diagnosis in patients with a remote history of pelvic radiation. A high index of suspicion is essential in cases of unexplained ascites, acute kidney injury, and urinary abnormalities. Early recognition and surgical intervention are crucial for favorable outcomes.

## Introduction

Ascites refers to the pathological accumulation of fluid in the peritoneal cavity, which may result in abdominal distension and discomfort. Associated symptoms may include anorexia, nausea, early satiety, heartburn, flank pain, and respiratory distress as fluid volume increases. Cirrhosis accounts for 80-84% of ascites cases. Other causes include chronic kidney disease, nephrotic syndrome, cardiac ascites, peritoneal carcinomatosis, abdominal malignancies, tuberculosis, and pancreatic disease [1]. Another possible, although rare, cause of ascites is the accumulation of urine due to bladder rupture [2].

Spontaneous rupture of the urinary bladder is a rare but serious medical emergency that can be fatal if not promptly diagnosed and treated [3].

The exact incidence of spontaneous bladder rupture as a late complication of radiation therapy remains unclear. Reported cases often associate this condition with underlying factors such as lower urinary tract obstruction, bladder lesions (e.g., tuberculosis), recurrent urinary tract infections or prolonged cystitis, bladder diverticula, necrosis caused by vascular events, bladder carcinoma, or complications following bladder surgery or pelvic radiation therapy [4].

Radiotherapy for pelvic cancers often affects nearby organs such as the bladder, ureters, and rectum, with side effects varying in severity. Clinically significant side effects from curative radiotherapy for primary pelvic carcinoma occur in approximately 10% of cases, with around 2-3% involving urological complications [3,5].

Common clinical signs include hypogastric pain or tenderness, abdominal distension, hematuria, and other non-specific symptoms. However, bladder rupture is rarely considered in the initial differential diagnosis, as these symptoms overlap with other conditions (such as peritonitis, pancreatitis, gastrointestinal perforation, and intestinal ischemia), potentially leading to delays in detection [6]. Diagnostic confirmation can be achieved through cystoscopy, fluoroscopic retrograde cystography, or computed tomography (CT) with retrograde cystography [6,7].

## Case presentation

A 51-year-old female presented to the emergency department (ED) with a two-week history of abdominal pain, accompanied by abdominal distension, asthenia, nausea, dysuria, and oliguria over the preceding week. One week earlier, she had been evaluated in the ED with the same symptoms and discharged with analgesics, as no abnormalities were detected in her laboratory tests.

The patient's medical history featured invasive cervical carcinoma treated with chemotherapy and radiotherapy 15 years earlier, with no evidence of recurrence. Other comorbidities included obesity, dyslipidemia, and depression.

Upon physical examination, the patient was tachycardic (heart rate: 122 bpm), afebrile, and normotensive (blood pressure: 124/71 mmHg), with normal peripheral oxygen saturation (SpO_2_: 98%). She exhibited altered mental status, presenting with disorientation and psychomotor retardation, with a Glasgow Coma Scale score of 13. The abdomen was distended, with a palpable fluid thrill. It was painful upon deep palpation but with no rebound tenderness.

**Figure 1:**
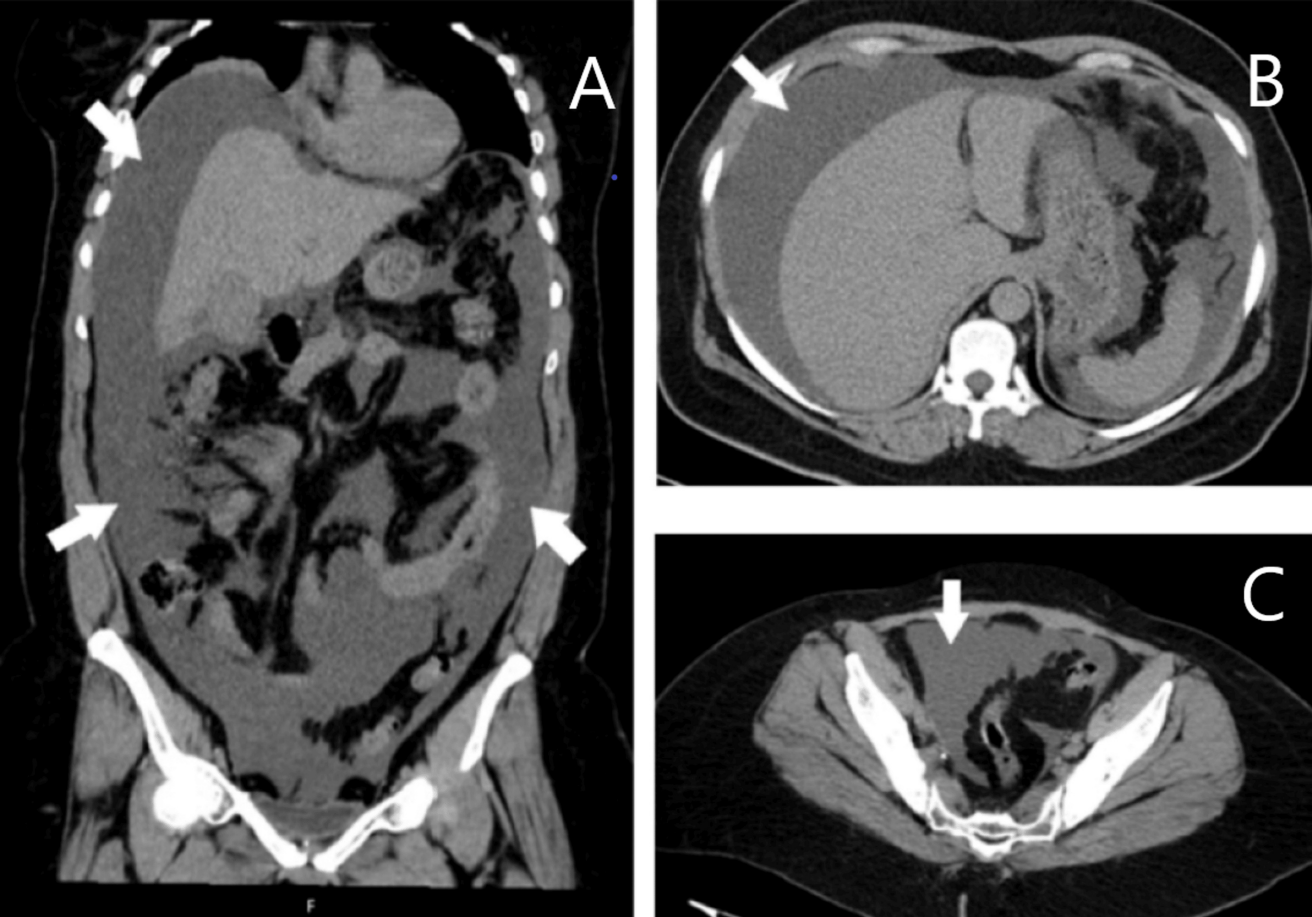
CT scan findings on admission Abdominal CT showing moderate-to-large-volume ascites (marked by the white arrows). Sub-image A displays the coronal plane. Sub-images B and C depict the axial plane.

Laboratory findings revealed leukocytosis, elevated C-reactive protein, acute kidney injury with a serum creatinine level of 8.21 mg/dL (compared to 0.91 mg/dL the previous week), metabolic acidosis, and hyperkalemia (see Tables 1, 2). A computed tomography (CT) scan was performed to help clarify the cause of abdominal pain and rule out an obstructive cause for acute kidney injury. It showed moderate-to-large volume ascites, an empty bladder, and no other significant abnormalities (Figure 1).

**Table 1:** Main laboratory test results in the emergency department

Laboratory test	Result	Reference value
Hemoglobin (g/gL)	17.8	12.0-16.0
White blood cells	15.0 x10^3^/μL	4.8-10.8 x10^3^/μL
Neutrophils	13.4 x10^3^/μL	1.8-7.7 x10^3^/μL
Eosinophils	0.0 x10^3^/μL	0.00-0.49 x10^3^/μL
Basophills	0.0 x10^3^/μL	0.0-0.1 x10^3^/μL
Lymphocytes	0.7 x10^3^/μL	1.0-4.8 x10^3^/μL
Monocytes	0.8 x10^3^/μL	0.12 – 0.80 x10^3^/μL
Platelets	594 x10^3^/μL	150-350 x10^3^/μL
C-reactive protein (mg/L)	58.6	<3.0
Urea (mg/dL)	247	15-39
Creatinine (mg/dL)	8.21	0.57-1.11

**Table 2:** Arterial blood gas analysis in the emergency department Ca²⁺: calcium ion concentration; Cl⁻: chloride ion concentration; HCO₃⁻: bicarbonate concentration; K⁺: potassium ion concentration; Na⁺: sodium ion concentration pCO₂: partial pressure of carbon dioxide; pO₂: partial pressure of oxygen

Arterial blood gas analysis	Result	Reference value
pH	7,251	7.350-7.450
pCO^2 ^(mmHg)	25.1	35.0-45.0
pO^2 ^(mmHg)	91.0	80.0-100.0
HCO^3-^ (mmol/L)	9.9	22.0-31.0
K^+ ^(mmol/L)	6.53	3.50-5.10
Na^+ ^(mmol/L)	123.9	135-145
Cl^- ^(mmol/L)	96.0	87.0-106.0
Ca^2+ ^(mmol/L)	1.31	1.15-1.35

A urinary catheter was placed for more effective monitoring of the urinary output. A urine sample was collected and the urinalysis revealed leukocyturia (Table 3).

**Table 3:** Urinalysis in the emergency department

Urinary analysis	Result	Reference value
Density	1.025	
Leucocytes (per mcl)	1702	<28
Erytrocytes (per mcl)	96	<20
Hyaline cilinders (per mcl)	15	<4

Paracentesis was initially planned to analyze the ascitic fluid and attempt to identify its cause, but during preparation, the patient's abdomen became softer and less painful, and shifting dullness was less evident. Notably, despite an empty bladder in the image, 3 L of fluid was drained within one hour, without hemodynamic repercussions, which led us to question the origin of this fluid.

**Figure 2:**
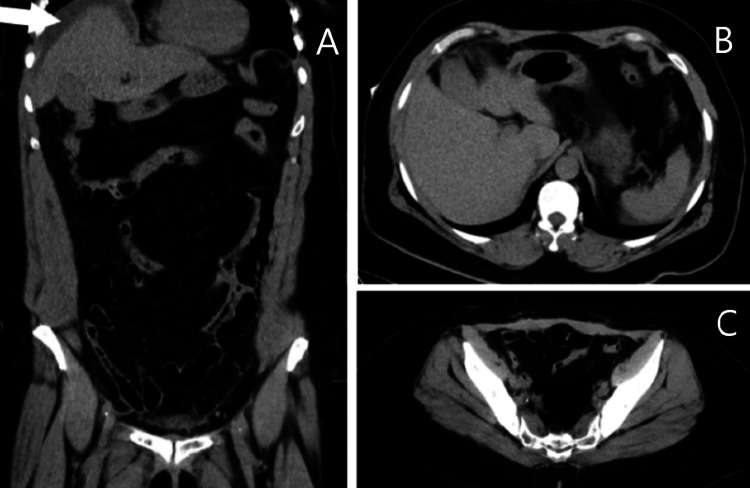
Follow-up CT scan after catheterization Abdominal CT after catheterization, showing a reduction in the ascitic fluid (marked by the white arrow). Sub-image A shows the coronal plane. Sub-images B and C show the axial plane.

This raised suspicion of bladder rupture, and a repeat CT scan (since ultrasonography was unavailable) showed a notable reduction in ascitic fluid volume compared to the previous scan, although it did not conclusively confirm bladder rupture (Figure 2).

**Figure 3:**
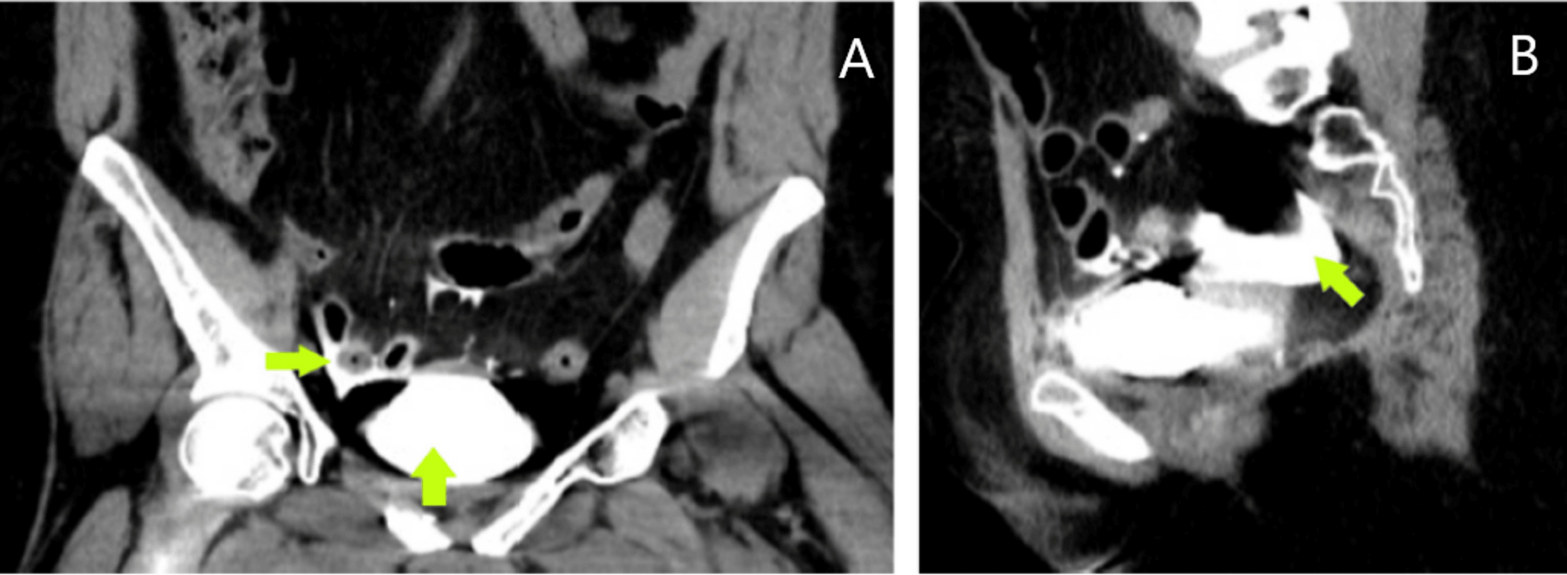
Follow-up CT scan after the administration of contrast fluid through the urinary catheter Abdominal CT showing intraperitoneal extravasation of contrast fluid (marked by the green arrows). Sub-image A shows the coronal plane, while sub-image B shows the sagittal plane.

Consequently, contrast was injected through the urinary catheter, and a follow-up CT scan revealed an intraperitoneal bladder rupture (Figure 3).

Broad-spectrum antibiotics were initiated, and the patient was transferred to the urology ED. She underwent abdominal laparotomy, during which a 0.5 cm bladder tear was repaired. The patient recovered well and was discharged 10 days later.

## Discussion

Spontaneous bladder rupture is a rare condition, occurring in less than 1% of cases, with an estimated incidence of one in 126,000 people. Trauma accounts for the majority of bladder ruptures (96%) [8]. Spontaneous or iatrogenic ruptures typically involve the intraperitoneal region, whereas traumatic ruptures, especially those associated with pelvic fractures, are more often extraperitoneal. The bladder is most vulnerable to rupture when distended, with the dome being the weakest and most commonly affected site [7].

Radiation therapy can induce a variety of pathological changes in the bladder, including inflammation, fibrosis, cellular atypia, and necrosis, which can affect all layers of the bladder wall and its vasculature [9]. These changes weaken the bladder structurally, increasing the risk of spontaneous perforation. These effects depend on factors such as the radiation dose, fractionation, and the volume of tissue irradiated [4].

In adults, spontaneous bladder perforations have been reported as late as 15 years after radiation therapy [3]. Recurrent episodes of perforation are possible and should be considered in acute cases.

The patient was oliguric, with high levels of serum creatinine and hyperkalemia, which led us to consider acute kidney injury. In fact, this was a case of “pseudo-renal failure." Urinary ascites caused by bladder rupture contains fluid rich in creatinine. The higher concentration of urinary waste products in the peritoneal cavity compared to the bloodstream establishes a concentration gradient that promotes the reabsorption of azotemic substances into the blood. This process leads to electrolyte disturbances, such as hyperkalemia, and apparent renal dysfunction. However, the biochemical abnormalities observed do not reflect true renal impairment but instead result from reverse dialysis across the peritoneal membrane [10,11].

The differential diagnosis of ascites with acute kidney injury includes common etiologies such as cirrhosis and nephrotic syndrome, which can cause transudative ascites due to hypoalbuminemia, and malignancy [12], but other causes must also be considered. Similarly, spontaneous rupture of intra-abdominal organs, such as the spleen in hematologic or other malignancies, can present with sudden abdominal pain and hemoperitoneum, leading to initial diagnostic uncertainty [13,14]. Other important considerations include pancreatic ascites due to chronic pancreatitis [15,16] and chylous ascites from lymphatic obstruction [17,18]. However, in this case, the rapid resolution of ascites following catheterization and confirmation of intraperitoneal contrast extravasation on imaging established the final diagnosis of spontaneous bladder rupture.

This case highlights the diagnostic challenge of spontaneous bladder rupture in patients with a history of pelvic radiation therapy. It emphasizes the importance of integrating clinical history, risk factors, and appropriate diagnostic tools, such as repeat imaging and CT cystograms, in confirming the diagnosis and guiding timely surgical intervention to prevent complications associated with bladder rupture, such as sepsis and electrolyte imbalances.

## Conclusions

Spontaneous bladder rupture is a rare and potentially life-threatening condition. Clinical history and risk factor identification are essential, particularly in patients with ascites of unclear etiology.

A CT cystogram is a highly sensitive diagnostic tool and can be readily performed in emergency settings. Treatment involves drainage of effused urine, surgical repair of the bladder perforation, and broad-spectrum antibiotic therapy to prevent complications.

Clinicians should remain vigilant for bladder rupture in patients with risk factors such as prior pelvic radiation therapy, chronic urinary retention, or recurrent infections, as a delayed diagnosis can significantly worsen outcomes. This case underscores the importance of considering rare diagnoses in complex presentations to ensure optimal patient care.
